# Development and Assessment of Nomogram Based on AFP Response for Patients with Unresectable Hepatocellular Carcinoma Treated with Immune Checkpoint Inhibitors

**DOI:** 10.3390/cancers15215131

**Published:** 2023-10-25

**Authors:** Yi Zhang, Hui Shen, Ruiying Zheng, Yueting Sun, Xiaoyan Xie, Ming-De Lu, Baoxian Liu, Guangliang Huang

**Affiliations:** 1 Division of Interventional Ultrasound, Department of Medical Ultrasonics, Institute of Diagnostic and Interventional Ultrasound, The First Affiliated Hospital of Sun Yat-sen University, 58 Zhong Shan Road 2, Guangzhou 510080, China; zhangy2235@mail2.sysu.edu.cn (Y.Z.); shenh25@mail2.sysu.edu.cn (H.S.); lumd@live.com (M.-D.L.); 2 Department of Hepatobiliary Surgery, The First Affiliated Hospital of Sun Yat-sen University, 58 Zhong Shan Road 2, Guangzhou 510080, China; 3 Department of Medical Ultrasonics, Guangxi Hospital Division, The First Affiliated Hospital of Sun Yat-sen University, Nanning 530022, China

**Keywords:** hepatocellular carcinoma, immune checkpoint inhibitor, AFP response, nomogram, progression-free survival

## Abstract

**Simple Summary:**

Immune checkpoint inhibitors (ICIs) have been increasingly used to treat hepatocellular carcinoma (HCC) but lack effective biomarkers. Our study aimed to investigate the related factors affecting the efficacy of ICIs treatment and develop a prognostic nomogram for patients with unresectable HCC receiving ICIs therapy. The nomogram may predict the treatment efficacy and help decision making in daily clinical practice.

**Abstract:**

Background: Immune checkpoint inhibitors (ICIs) have been increasingly used to treat hepatocellular carcinoma (HCC). Prognostic biomarkers are an unmet need. We aimed to develop a prognostic nomogram for patients with unresectable HCC receiving ICIs therapy. Methods: A total of 120 patients with unresectable HCC receiving ICIs treatment were enrolled in this study. Patients were randomly divided into a training set (n = 84) and a validation set (n = 36) in a 7:3 ratio. Clinical characteristics were retrospectively analyzed. Serum α-fetoprotein protein (AFP) response was defined as a decline of ≥20% in AFP levels within the initial eight weeks of treatment. Univariable and multivariable Cox analyses were used to select relevant variables and construct the nomogram. The areas under the receiver operating characteristic curves (AUCs) were used to determine the performance of the model. Kaplan–Meier analysis with the log-rank test was used to compare different risk groups. Results: The median progression-free survival (PFS) was 7.7 months. In the multivariate Cox analysis, the presence of extrahepatic metastasis (hazard ratio [HR] = 2.08, 95% confidence interval [CI]: 1.02–4.27, *p* < 0.05), white blood cell count (HR = 3.48, 95% CI: 1.02–11.88, *p* < 0.05) and AFP response (HR = 0.41, 95% CI: 0.18–0.95, *p* < 0.05) independently predicted PFS. A nomogram for PFS was established with AUCs of 0.79 and 0.70 in the training and validation sets, respectively. The median PFS of the high- and low-risk subgroups was 3.5 and 11.7 months, respectively (*p* < 0.05). Conclusion: The nomogram could predict PFS in patients with unresectable HCC receiving ICIs treatment and further help decision making in daily clinical practice.

## 1. Introduction

Hepatocellular carcinoma (HCC) is the sixth most common malignancy and the third leading cause of cancer death worldwide [1]. A majority of patients present with intermediate and advanced-stage disease, with poor prognosis [2,3]. The Barcelona Clinic Liver Cancer (BCLC) staging system is the most widely used treatment algorithm worldwide. The BCLC staging and treatment strategy, in 2022, stated that for advanced-stage HCC and intermediate stage patients with diffuse, infiltrative, extensive HCC liver involvement, systemic therapy should be the recommended option [2].

In 2007, the SHARP experiment found that sorafenib monotherapy is significantly better than placebo for advanced HCC. Sorafenib obtained approval from the United States Food and Drug Administration (FDA) and dominated drug treatment for over a decade [4]. In 2018, the REFLECT phase III clinical study showed that the overall survival (OS) of patients treated with lenvatinib was not inferior to that of sorafenib. The lenvatinib was approved for first-line FDA treatment, breaking the dominance of sorafenib and becoming a new treatment option for advanced HCC [5]. In recent years, various internationally recognized therapeutic choices for HCC have expanded rapidly, and immune checkpoint inhibitors (ICIs) have been extensively investigated in patients with HCC. Unlike traditional treatment methods, ICIs do not directly destroy tumor cells but rather activate immune cells by targeting the immune system or tumor microenvironment to kill tumors [6,7]. The Phase I/II Checkmate040 clinical study showed that the median OS and objective response rate of the treatment group with nivolumab were significantly better than those with sorafenib for first-line treatment of advanced HCC. Nivolumab became the first immunotherapy drug for HCC approved by the FDA for second-line treatment, marking the beginning of the era of immunotherapy for advanced HCC [8]. There has already been one anti-programmed cell death protein (PD)-1 (pembrolizumab) with two combinations, namely, nivolumab-ipilimumab regimen and atezolizumab-bevacizumab regimen, approved by the United States Food and Drug Administration [9]. Compared with sorafenib, the combination of atezolizumab with bevacizumab significantly improved the median OS and progression-free survival (PFS) of patients: 19.2 vs. 13.4 months (HR = 0.66, *p* < 0.001) and 6.9 months vs. 4.3 months (HR = 0.65, *p* < 0.001) [10], making this combination the benchmark for first-line systemic treatment for the majority of HCC patients [2].

However, the objective response rate (ORR) for ICI monotherapy ranged from 15% to 23% and only increased to about 30% after combination therapy [7]. Several predictive biomarkers for ICIs treatment have been proposed, including PD-L1 expression, tumor mutational burden (TMB), microsatellite instability status (MSI) and activated Wnt/β-catenin signaling [11,12,13,14,15]. However, these biomarkers can only be acquired from tumor tissues, and the utility is limited because of the invasive procedures. Serum alpha-fetoprotein (AFP) is a glycoprotein acquired from a simple blood test routinely available in clinical practice. AFP is the most widely accepted serum biomarker in the surveillance and non-invasive diagnosis of HCC. Dynamic changes in AFP levels are associated with the therapeutic effect of HCC and guide clinical practice in most cases [16]. Previous studies have shown that early AFP response was associated with therapeutic efficacy and prognosis of ICIs therapy for HCC [17,18,19]. A systematic review with meta-analysis revealed that a decline of ≥20% in AFP levels within eight weeks of treatment may be the appropriate definition for early AFP response [20]. A comprehensive prognostic model, including AFP response, could be developed to predict the survival outcomes and guide treatment decision making of ICIs therapy for unresectable HCC.

Therefore, this study explored potential predictors associated with the survival of ICIs treatment and developed a prognostic nomogram based on early AFP response in patients with unresectable HCC.

## 2. Materials and Methods

The study was approved by the Ethics Committee of the First Affiliated Hospital of Sun Yat-Sen University (Guangzhou, China). Since it was a retrospective study, informed consent from the patients could not be obtained, but all the patients had signed informed consent before the ICIs treatment.

### 2.1. Patients

**Figure 1:**
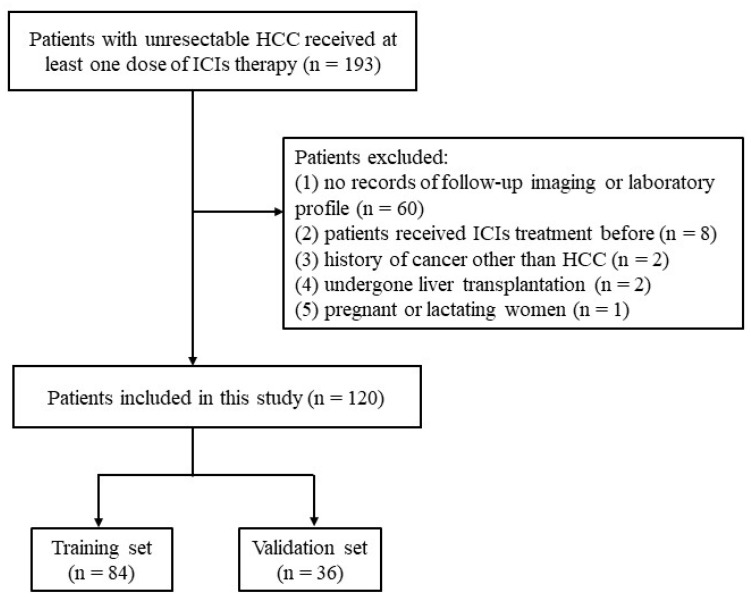
Patient selection flowchart.

From January 2019 to January 2023, a total of 193 patients with unresectable HCC received ICIs treatment and were enrolled in this study. The inclusion criteria were as follows: (1) age more than 18 years old; (2) pathologically or clinically diagnosed with HCC following the European Association for the Study of the Liver [21]; (3) patients with HCC who were not suitable for curative treatment (resection, transplantation or ablation) and received at least one dose of ICI therapy; (4) Child-Pugh class A or B; (5) Eastern Cooperative Oncology Group performance status (ECOG PS) <2. The exclusion criteria were as follows: (1) no records of follow-up imaging or laboratory profile (n = 60); (2) patients who received ICIs treatment before (n = 8); (3) history of cancer other than HCC (n = 2); (4) patients who underwent liver transplantation (n = 2); (5) pregnant or lactating women (n = 1). We finally included 120 patients in this study (Figure 1). The patients were randomly divided into training (n = 84) and validation cohorts (n = 36) at a ratio of 7:3. The randomization process was performed once with the “caret” package of R studio.

Clinical data of patients were acquired from electronic medical records, mainly including gender, age, etiology (hepatitis B virus infection), combination therapy (transarterial chemoembolization (TACE) or hepatic artery infusion chemotherapy (HAIC)), Child-Pugh class and Barcelona Clinic Liver Cancer (BCLC stage). In addition, imaging profile (tumor number, max tumor size, portal vein invasion, extrahepatic metastasis) and laboratory parameters were acquired before the start of treatment, including peripheral blood count (platelet (PLT), white blood cell count (WBC), neutrophil count (NEUT), monocyte count (MONO)), liver function (albumin (ALB), alanine aminotransferase (ALT), total bilirubin (TBIL), prothrombin time (PT)) and tumor marker (AFP). We further collected AFP levels within the initial eight weeks after ICIs treatment. NLR (absolute neutrophil count/absolute lymphocyte count), PLR (absolute platelet count/absolute lymphocyte count) and MLR (absolute monocyte count/absolute lymphocyte count) were calculated.

### 2.2. Treatment Strategy for HCC

Patients were evaluated by a multidisciplinary team consisting of expert radiologists, interventional radiologists, oncologists and surgeons in the field of HCC in the First Affiliated Hospital of Sun Yat-sen University for treatment guidance based on the tumor burden and general condition. ICIs treatment was recommended based on the status and general condition of the HCC. ICIs were intravenously administered at the standard dose every three weeks: camrelizumab 200 mg, sintilimab 200 mg, tislelizumab 200 mg, toripalimab 240 mg and pembrolizumab 200 mg. Patients were treated according to the treatment plan until intolerable adverse events or disease progression.

### 2.3. Follow-up

Patients underwent abdominal contrast-enhanced computer tomography (CT), magnetic resonance imaging and chest-enhanced CT every 6–8 weeks during treatment. Tumor response was assessed according to Modified Response Evaluation Criteria in Solid Tumors (mRECIST). Progressive disease (PD) was defined as an increase of at least 20% in the sum of the diameters of viable target lesions in the arterial phase, taking as reference the baseline value or if new lesions appeared [22]. We further collected AFP levels within the initial eight weeks after ICI treatment. Based on previous studies, AFP response was defined as a decline of ≥20% in the serum AFP level within eight weeks of ICI therapy [17,18,20,23]. PFS was defined as the time from the start of the first dose of ICI therapy to radiologic tumor progression, death or the last follow-up.

### 2.4. Statistical Analysis

Continuous variables were expressed as medians (interquartile range, IQR) and were compared by the Mann–Whitney U test. Categorical variables were expressed as numerical values with percentages and were compared by chi-squared or Fisher’s exact tests. We included 23 variables that could be classified as tumor burden, pretreatment laboratory inflammatory indicator and AFP response after treatment and used Cox regression analysis to identify factors associated with PFS. Variables with a *p* value less than 0.05 on the univariable analysis were considered for multivariable Cox analysis to construct the nomogram in the training set. The nomogram constructed from the training set was evaluated in the validation sets. The areas under the receiver operating characteristic curves (AUCs) were used to determine the sensitivity and specificity of the nomogram. Kaplan–Meier analysis with the log-rank test was used to compare different risk groups. All analyses were performed using R software (version 4.1.1, Chicago, IL, USA). A two-sided *p* value less than 0.05 was considered statistically significant. The software packages involved in this study were “ggplot2”, “survminer”, “survival”, “glmnet”, “rms”, “riskRegression”, “dcurves”, “caret” and “nomogramFormula”.

## 3. Results

### 3.1. Patient Characteristics

The median age of this cohort was 52 years (IQR, 42–61). One hundred and sixteen (96.7%) patients were infected with the hepatitis B virus. Ninety-five patients (79.2%) had Child-Pugh A liver function, and 101 (84.2%) patients were in BCLC stage C. Overall, 87 (72.5%) had intrahepatic tumors >3, and 76 (63.3%) had a maximum tumor size >5 cm. Sixty-one (50.8%) patients had portal vein invasion, and 71 (59.2%) had extrahepatic metastases. The baseline AFP level of 48.3% (58/120) of the patients was beyond 400 μg/L, and 48.3% (58/120) detected AFP response. Ninety-six (80%) patients received a combination of transarterial chemoembolization or hepatic artery infusion chemotherapy. Until the last follow-up, the median PFS (mPFS) was 7.7 months. More patients with multiple tumors were in the validation (88.9%) than in the training set (65.5%), and the difference was statistically significant. The mPFS of the training and validation sets was 4.5 months and 5.5 months, respectively, with no statistical difference between the two groups (*p* = 0.82). The mPFS distribution of the two groups was shown in Figures S1 and S2. The baseline data, including gender, age, HBV infection status, max tumor size, portal vein invasion, extrahepatic metastasis, Child-Pugh class, BCLC stage and combination therapies, PLT, ALB, ALT, TBIL, PT, WBC, NEUT, MONO, AFP, NLR, PLR and MLR, did not show a statistically significant difference between the training set and the validation set. The detailed baseline characteristics of 120 patients are listed in Table 1.

### 3.2. Independent Predictors of PFS and Predictive Nomogram Construction (Training Set)

**Figure 2:**
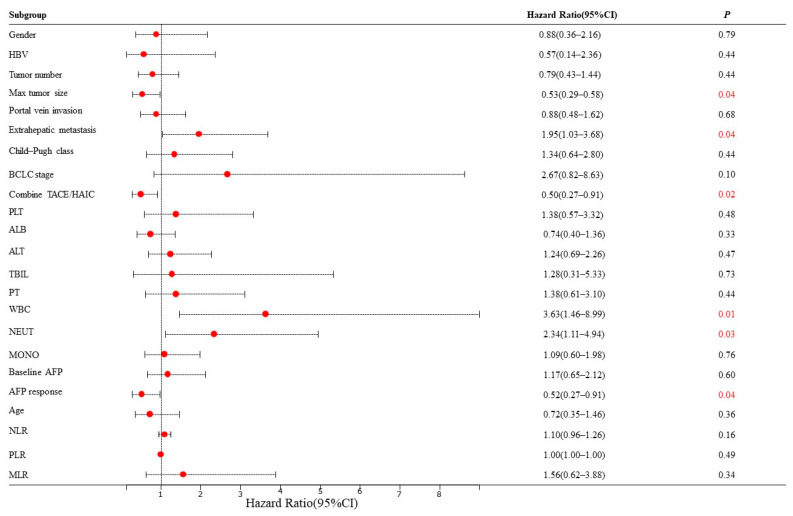
Univariate Cox regression analyses for PFS of HCC patients in the training set. The red number represents a *p* value less than 0.05. HBV: hepatitis B virus; BCLC: Barcelona Clinic Liver Cancer; TACE: transarterial chemoembolization; HAIC: hepatic artery infusion chemotherapy; PLT: platelet; ALB: albumin; ALT: alanine aminotransferase; TBIL: total bilirubin; PT: prothrombin time; WBC: white blood cell count; NEUT: neutrophil; MONO: monocyte; AFP, alpha-foetoprotein; NLR: neutrophil-to-lymphocyte ratio neutrophil; PLR: platelet-to-lymphocyte ratio; MLR: monocyte–to-lymphocyte ratio.

**Figure 3:**
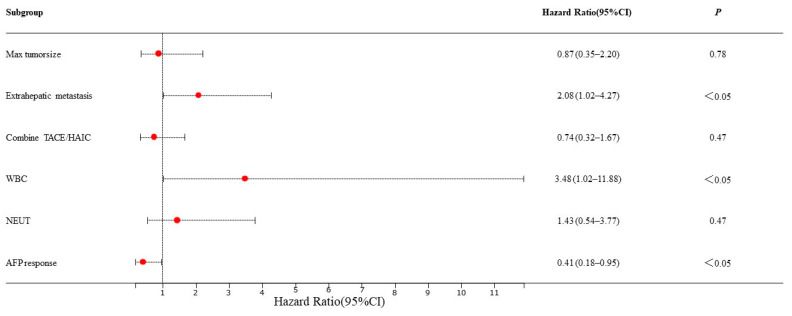
Multivariate Cox regression analyses for PFS of HCC patients in the training set. TACE: transarterial chemoembolization; HAIC: hepatic artery infusion chemotherapy; WBC: white blood cell count; NEUT: neutrophil.

**Figure 4:**
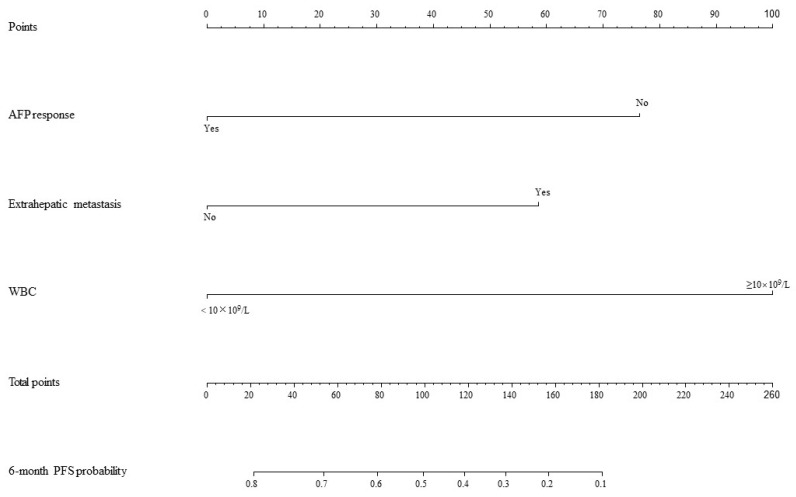
Nomogram for PFS. AFP: alpha-foetoprotein; WBC: white blood cell count; PFS: progression-free survival.

Based on the mRECIST guidelines, PD was observed in 46 (54.8%) patients, and the mPFS was 4.5 months in the training set. In the univariate analysis, a max tumor size >5 cm (HR = 0.53, 95% CI: 0.29–0.58, *p* = 0.04), presence of extrahepatic metastasis (HR = 1.95, 95% CI: 1.03–3.68, *p* = 0.04), combined TACE /HAIC (HR = 0.50, 95% CI: 0.27–0.91, *p* = 0.02), WBC ≥ 10 × 10^9^/L (HR = 3.63, 95% CI: 1.46–8.99, *p* = 0.01), NEUT ≥ 6.4 × 10^9^/L (HR = 2.34, 95% CI: 1.11–4.94, *p* = 0.03) and AFP response (HR = 0.52, 95% CI: 0.27–0.91, *p* = 0.04) were associated with PFS (Figure 2). These predictors were included in the multivariate analysis. The presence of extrahepatic metastasis (HR = 2.08, 95% CI: 1.02–4.27, *p* < 0.05), WBC ≥ 10 × 10^9^/L (HR = 3.48, 95% CI: 1.02–11.88, *p* < 0.05) and AFP response (HR = 0.41, 95% CI: 0.18–0.95, *p* < 0.05) independently predicted PFS (Figure 3). Following that, a nomogram with independent predictors of PFS based on extrahepatic metastasis, WBC and AFP response was constructed to evaluate the 6-month PFS probability (Figure 4).

### 3.3. Model Performance and Validation

**Figure 5:**
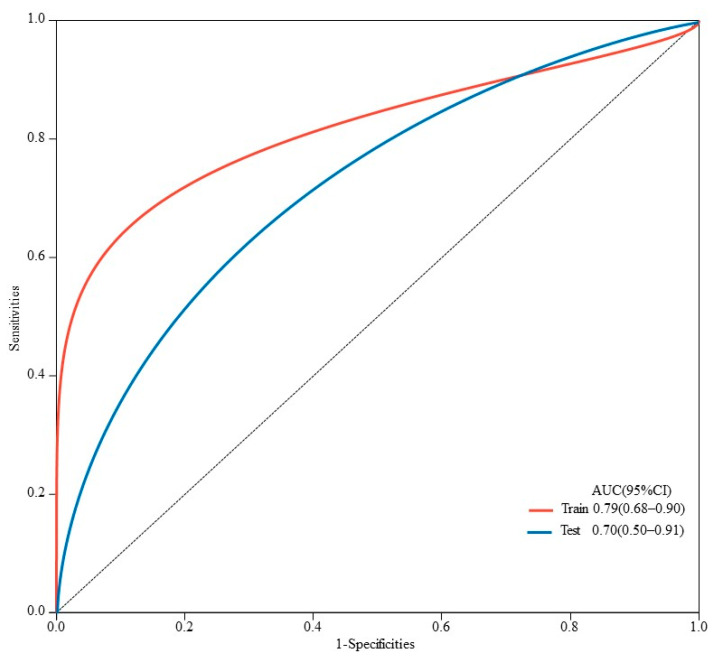
ROC curves for predicting PFS in HCC patients in training and validation sets. AUC is 0.79 (95% CI, 0.90–0.68) in the training set and 0.70 (95% CI, 0.91–0.50) in the validation set.

**Figure 6:**
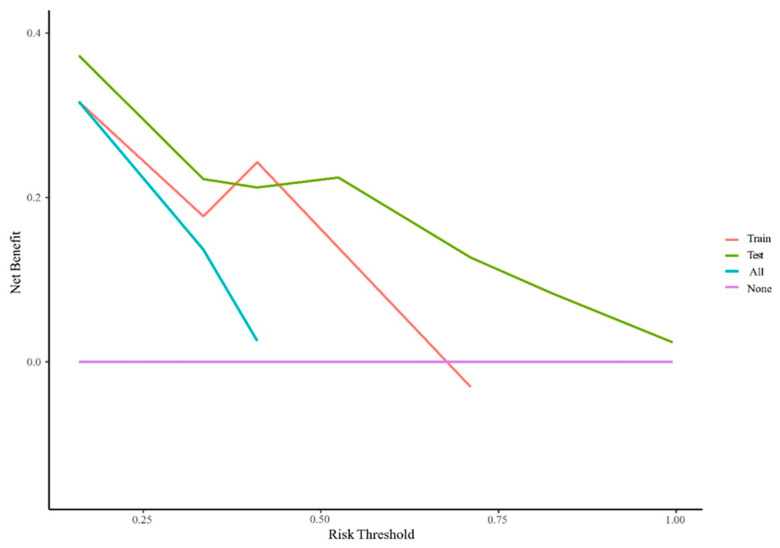
Decision curve analysis for PFS in the training cohort. The Y-axis is the net benefit (NB), and the X-axis is the risk threshold probability. The red and green lines belong to the NB of the prediction model in the training and validation set. The blue line is the NB when all patients are assumed to be treated, and the purple line is the NB when none are assumed to be treated.

**Figure 7:**
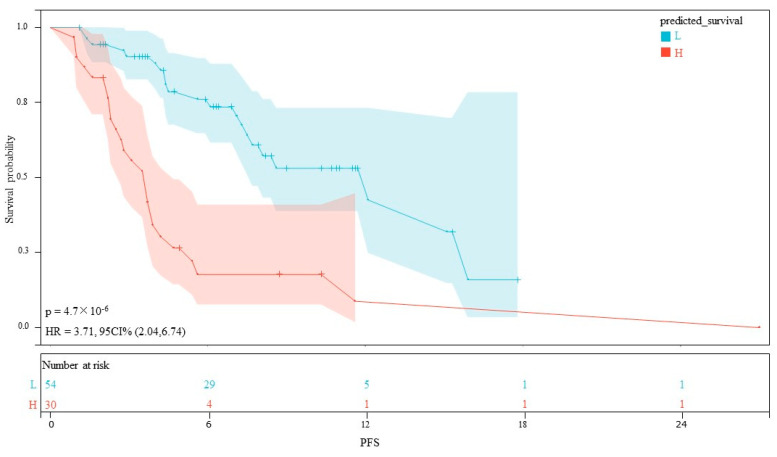
Kaplan–Meier curves for PFS between the high- and low-risk groups.

The AUC values for AFP response, metastasis and WBC were 0.59 (95% CI, 0.53–0.65), 0.66 (95% CI, 0.60–0.71) and 0.59 (95% CI, 0.55–0.62), respectively. The AUC values of the multivariable models were 0.79 (95% CI, 0.90–0.68) and 0.70 (95% CI, 0.91–0.50) for the training set and the validation set, respectively (Figure 5). Decision curve analysis (DCA) showed that the models predicted a clear net benefit compared to “none” or “all”, and the clinical utility was better (Figure 6). According to the constructed model, the study cohort was divided into high-risk and low-risk groups, with mPFS of 3.5 months and 11.7 months, respectively. The cutoff value for low-risk and high-risk groups is 0. Kaplan–Meier curves among groups were shown in Figure 7, with significant differences between groups (HR = 3.71, 95CI%: 2.04–6.74, *p* < 0.05).

## 4. Discussion

The results of this study revealed that AFP response, extrahepatic metastasis and WBC count were independent predictors of PFS in patients with unresectable HCC receiving ICIs therapies. We included three variables and developed a prognostic model that exhibited good discrimination abilities in the training set and achieved good verification results in the validation set. The model could help identify patients who may benefit from ICIs therapies.

The advent of immunotherapy has revolutionized the landscape of cancer treatment, offering new hope for patients with advanced HCC. However, no reliable biomarkers have been found to predict the efficacy of immunotherapy for HCC. The expression status of PD-L1, TMB, MSI and specific gene mutations are widely studied predictive indicators for the therapeutic efficacy of ICIs. However, the proportion of HCC with high TMB and MSI is deficient. A study analyzed the genomic profiling of 755 HCC specimens; the TMB-high only accounted for 1%. Of 542 cases assessed for MSI, only one MSI-high and TMB-high were found [12]. In addition, the expression of PD-L1 is generally low, accounting for approximately 10–20% of tumor cells [24]. Although some clinical studies suggest that patients with PD-L1 expression can benefit from immunotherapy, there is little difference with PD-L1 negative HCC, and objective response can also be observed in PD-L1 negative HCC [8]. Different detection methods, spatial heterogeneity and standard thresholds further limit the exploration and application of the above biomarkers [25,26]. A simple and non-invasive evaluation and prediction method is urgently demanded in current clinical practice.

AFP level is widely used in the surveillance and non-invasive diagnosis of HCC, and its predictive value on the prognosis of HCC has also been reported [27,28,29]. In addition to promoting tumor growth by several mechanisms that include apoptotic regulation and cytoplasmic signaling modulation, AFP may also mediate suppression of the anti-tumor immune response, as it interacts with macrophages to decrease their phagocytic activity, inhibits the activity of natural killer cells, reduces proliferation of T-lymphocytes and promotes the activity of T-suppressor cells [30]. Moreover, previous studies have demonstrated that AFP may stimulate angiogenesis and induce metastasis of HCC. Increased serum AFP concentration was correlated with the up-regulation of vascular endothelial growth factor (VEGF) signaling in HCC tissue [31]. Excessive levels of VEGF can promote angiogenesis and induce an immunosuppressive tumor microenvironment [32]. The tumor growth-promoting and immunosuppressive activities of AFP may hamper the efficacy of immunotherapy.

Increasing studies have demonstrated that a decline in AFP levels is a positive predictor of ICIs treatment. Lee et al. proposed a 10-10 rule to early predict ICI response and OS based on baseline AFP levels ≥10 ng/mL and a 10% reduction within four weeks of treatment [19]. Hsu et al. reported that a ≥20% decline in AFP levels within the first three months of treatment predicted objective response and PFS [18]. Shao et al. reported that a >20% decline in AFP levels within the first four weeks of treatment was associated with higher efficacy of ICIs for advanced HCC [17]. A previous study revealed that a decline of ≥20% in AFP levels within eight weeks of treatment may be the appropriate definition for early AFP response [20]. In this study, we constructed a prognostic model based on AFP response for unresectable HCC receiving ICIs therapies.

According to the BCLC stage, BCLC C includes patients presenting with vascular invasion or extrahepatic metastasis and should be evaluated for systemic therapies [2]. Extrahepatic metastasis is a significant independent prognostic factor for ICIs therapy in unresectable HCC, which is consistent with the results of the previous study [33]. Unlike traditional chemotherapy and molecular targeted therapy, ICIs induce anti-tumor effects by reactivating exhausted T cells and thus stimulating anti-tumor immunity. A previous study revealed that tumor response to ICIs in HCC may vary among different organs because heterogeneous tumor-immune microenvironments and lung metastases were shown to respond most favorably to ICIs [34]. Our study did not analyze different organs, and further research on specific metastatic sites is warranted.

Previous studies have evaluated the role of inflammatory markers, including serum C-reactive protein, NLR and PLR, as prognostic predictors for several malignancies treated with ICIs therapy [35,36,37]. The prognostic and predictive value of circulating neutrophils has been reported as an independent indicator or as part of the NLR [38]. In addition, higher levels of WBC count and NLR before treatment are independent predictors of poor survival in patients receiving chemotherapy for advanced colorectal cancer [39]. Our study reported that a circulating neutrophil level was associated with PFS in the univariate analysis, and a high WBC count negatively predicted PFS. These results may be due to the tumor-promoting effects, including fostering of cancer cell proliferation, metastatic seeding and angiogenesis induced by the inflammatory tumor microenvironment [40]. Circulating white cells increased during cancer-mediated myelopoiesis, with neutrophils the most abundant. The neutrophil precursors, including myelocytes and promyelocytes, might be released in an inflammatory environment. The elevation of neutrophils induces the production of neutrophil-derived cytokines such as VEGF, matrix metalloproteinases and interleukin-18 (IL-18). The metalloproteinases increase migration and extravasation, and IL-18 impairs natural killer cell and T cell function, thus promoting progression and metastasis [41].

Although many studies regarded NLR as a strong predictive prognostic factor, some reported negative results. For example, previous research reported that NLR was not an independent prognosticator of PFS in patients treated with atezolizumab plus bevacizumab [42]. In our study, neither neutrophils nor NLR were significantly associated with PFS. Due to our study’s relatively small sample size, we will further increase the sample size to verify this result.

Several studies have constructed prognostic models to predict the treatment efficacy of ICIs therapies. A previous study demonstrated that C-reactive protein and AFP score in immunotherapy (CRAFITY) are associated with survival and radiological response [35]. Hsu WF et al. combined the CRAFITY score and AFP response to predict treatment outcomes in patients with unresectable hepatocellular carcinoma receiving immunotherapy [43]. Chen Q et al. identified tumor burden, laboratory indicators and immune target-related adverse events as key factors associated with tumor response and constructed prognostic models for predicting the tumor response in HCC patients receiving immunotherapy combined with targeted therapy [44]. Our study considered tumor burden, pretreatment laboratory inflammatory indicator and AFP response after treatment as essential biomarkers for survival outcomes. Instead of focusing on a specific type of biomarker, our study further combined independent and different clinical factors to develop an objective scoring system. The indicators are non-invasive and typically discovered during clinical practice without additional costs. The prognostic model exhibited good specificity, accuracy and verification results, which could help clinicians identify patients who would benefit most from ICIs therapy.

There are some meta-analyses of relevant cohort studies to identify the prognostic factors for HCC patients undergoing ICI treatment. By conducting a meta-analysis of 47 relevant cohort studies and analyzing 18 risk factors, Ma D et al. identified AFP, albumin-bilirubin score (ALBI), NLR, ECOG performance status, ChildPugh stage, BCLC stage, tumor number and vascular invasion as predictors for the PFS model, with AUC values of 0.575, 0.749 and 0.691 at 1-year, 2-year and 3-year follow-up points [45]. Our study identified AFP response, WBC and metastasis as predictors for the PFS model, with AUCs of 0.79 and 0.70 at the 6-month time point in the training and validation sets. Our models demonstrated relatively good performance for the PFS prediction, but the follow-up time of our study is relatively short, and further follow-up is needed. Another meta-analysis included a total of 44 articles with 5322 patients and confirmed that only NLR, early AFP response (AFP decrease > 10–20% in 4–6 weeks after starting treatment) and ALBI grade were significantly associated with OS and PFS in HCC patients receiving ICIs [46]. Our study’s definition of AFP response is different, and further analysis to identify the optimal definition of AFP response is also warranted.

There were several limitations to our study. First, this is a retrospective study in a single center without external validation and with a small sample size of only 120 patients included. Second, most of the patients were infected with the hepatitis B virus. Our results should be interpreted with caution when investigating other populations. Third, the patients in this study were relatively mixed because the patients were treated with various ICIs agents. However, this may better represent the real-world HCC population. Finally, the follow-up time of this study is relatively short. Further follow-up is warranted to explore the predictors of OS. However, our study developed and validated the predictive nomogram for PFS, and these results can assist the early screening of patients who may benefit from ICIs treatment to avoid the risk of adverse reactions and improve clinical outcomes.

## 5. Conclusions

We constructed a comprehensive and simple nomogram for predicting PFS in patients with unresectable HCC receiving ICIs treatment. The model may predict the treatment efficacy and help decision making in daily clinical practice.

## Figures and Tables

**Table 1:** Baseline characteristics of patients in the training and validation sets (n = 120).

Variable	Training Set (n = 84)	Validation Set (n = 36)	*p*-Value
Gender			
Male	75 (89.29)	35 (97.22)	0.15
Female	9 (10.71)	1 (2.78)	
Age (year)			
<60	63 (75.00)	25 (69.44)	0.53
≥60	21 (25.00)	11 (30.56)	
HBV			
Negative	2 (2.38)	2 (5.56)	0.38
Positive	82 (97.62)	34 (94.44)	
Tumor number			
<3	29 (34.52)	4 (11.11)	0.01
≥3	55 (65.48)	32 (88.89)	
Max tumor size			
<5 cm	35 (41.67)	9 (25.00)	0.08
≥5 cm	49 (58.33)	27 (75.00)	
Portal vein invasion			
Negative	44 (52.38)	15 (41.67)	0.28
Positive	40 (47.62)	21 (58.33)	
Extrahepatic metastasis			
Negative	34 (40.48)	15 (41.67)	0.90
Positive	50 (59.52)	21 (58.33)	
Child-Pugh class			
A	66 (78.57)	29 (80.56)	0.81
B	18 (21.43)	7 (19.44)	
BCLC stage			
II	11 (13.10)	8 (22.22)	0.21
III	73 (86.90)	28 (77.78)	
Combine TACE/HAIC			
No	20 (23.81)	4 (11.11)	0.11
Yes	64 (76.19)	32 (88.89)	
PLT			
<100 × 10^9^/L	11 (13.10)	5 (13.89)	0.91
≥100 × 10^9^/L	73 (86.90)	31 (86.11)	
ALB			
<35 g/L	30 (35.71)	11 (30.56)	0.59
≥35 g/L	54 (64.29)	25 (69.44)	
ALT			
<40 U/L	46 (54.76)	21 (58.33)	0.72
≥40 U/L	38 (45.24)	15 (41.67)	
TBIL			
<34.2 μmol/L	80 (95.24)	33 (91.67)	0.44
≥34.2 μmol/L	4 (4.76)	3 (8.33)	
PT			
<14 s	75 (89.29)	33 (91.67)	0.69
≥14 s	9 (10.71)	3 (8.33)	
WBC			
<10 × 10^9^/L	76 (90.48)	34 (94.44)	0.47
≥10 × 10^9^/L	8 (9.52)	2 (5.56)	
NEUT			
<6.4 × 10^9^/L	72 (85.71)	34 (94.44)	0.17
≥6.4 × 10^9^/L	12 (14.29)	2 (5.56)	
MONO			
<0.5 × 10^9^/L	42 (50.00)	21 (58.33)	0.40
≥0.5 × 10^9^/L	42 (50.00)	15 (41.67)	
Baseline AFP			
<400 μg/L	43 (51.19)	19 (52.78)	0.87
≥400 μg/L	41 (48.81)	17 (47.22)	
AFP response			
No	45 (53.57)	17 (47.22)	0.52
Yes	39 (46.43)	19 (52.78)	
NLR	3.14 (2.41, 4.51)	2.90 (2.04, 4.13)	0.14
PLR	134.65 (96.17, 206.67)	154.62 (117.42, 199.42)	0.25
MLR	0.42 (0.31, 0.60)	0.41 (0.30, 0.47)	0.15

HBV: hepatitis B virus; BCLC: Barcelona Clinic Liver Cancer; TACE: transarterial chemoembolization; HAIC: hepatic artery infusion chemotherapy; PLT: platelet; ALB: albumin; ALT: alanine aminotransferase; TBIL: total bilirubin; PT: prothrombin time; WBC: white blood cell count; NEUT: neutrophil; MONO: monocyte; AFP: alpha-foetoprotein; NLR: neutrophil-to-lymphocyte ratio neutrophil; PLR: platelet-to-lymphocyte ratio; MLR: monocyte–to-lymphocyte ratio.

## Data Availability

The raw data supporting the conclusions of this article will be made available by the authors without undue reservation.

## Supplementary Materials

The following supporting information can be downloaded at: https://www.mdpi.com/article/10.3390/cancers15215131/s1, Figure S1: The mPFS distribution in the training set; Figure S2: The mPFS distribution in the validation set.

## References

[1] Sung H., Ferlay J., Siegel R.L., Laversanne M., Soerjomataram I., Jemal A., Bray F. (2021). Global Cancer Statistics 2020: GLOBOCAN Estimates of Incidence and Mortality Worldwide for 36 Cancers in 185 Countries. CA Cancer J. Clin..

[2] Reig M., Forner A., Rimola J., Ferrer-Fàbrega J., Burrel M., Garcia-Criado Á., Kelley R.K., Galle P.R., Mazzaferro V., Salem R. (2022). BCLC strategy for prognosis prediction and treatment recommendation: The 2022 update. J. Hepatol..

[3] Zhu A.X. (2006). Systemic therapy of advanced hepatocellular carcinoma: How hopeful should we be?. Oncologist.

[4] Llovet J.M., Ricci S., Mazzaferro V., Hilgard P., Gane E., Blanc J.F., de Oliveira A.C., Santoro A., Raoul J.L., Forner A. (2008). Sorafenib in advanced hepatocellular carcinoma. N. Engl. J. Med..

[5] Kudo M., Finn R.S., Qin S., Han K.H., Ikeda K., Piscaglia F., Baron A., Park J.W., Han G., Jassem J. (2018). Lenvatinib versus sorafenib in first-line treatment of patients with unresectable hepatocellular carcinoma: A randomised phase 3 non-inferiority trial. Lancet.

[6] Pinter M., Peck-Radosavljevic M. (2018). Review article: Systemic treatment of hepatocellular carcinoma. Aliment. Pharmacol. Ther..

[7] Pinter M., Jain R.K., Duda D.G. (2021). The Current Landscape of Immune Checkpoint Blockade in Hepatocellular Carcinoma: A Review. JAMA Oncol..

[8] El-Khoueiry A.B., Sangro B., Yau T., Crocenzi T.S., Kudo M., Hsu C., Kim T.Y., Choo S.P., Trojan J., Welling T.H.R. (2017). Nivolumab in patients with advanced hepatocellular carcinoma (CheckMate 040): An open-label, non-comparative, phase 1/2 dose escalation and expansion trial. Lancet.

[9] Chan S.L., Wong N., Lam W.K.J., Kuang M. (2022). Personalized treatment for hepatocellular carcinoma: Current status and future perspectives. J. Gastroenterol. Hepatol..

[10] Cheng A.L., Qin S., Ikeda M., Galle P.R., Ducreux M., Kim T.Y., Lim H.Y., Kudo M., Breder V., Merle P. (2022). Updated efficacy and safety data from IMbrave150: Atezolizumab plus bevacizumab vs. sorafenib for unresectable hepatocellular carcinoma. J. Hepatol..

[11] Zhu A.X., Finn R.S., Edeline J., Cattan S., Ogasawara S., Palmer D., Verslype C., Zagonel V., Fartoux L., Vogel A. (2018). Pembrolizumab in patients with advanced hepatocellular carcinoma previously treated with sorafenib (KEYNOTE-224): A non-randomised, open-label phase 2 trial. Lancet Oncol..

[12] Ang C., Klempner S.J., Ali S.M., Madison R., Ross J.S., Severson E.A., Fabrizio D., Goodman A., Kurzrock R., Suh J. (2019). Prevalence of established and emerging biomarkers of immune checkpoint inhibitor response in advanced hepatocellular carcinoma. Oncotarget.

[13] Sangro B., Melero I., Wadhawan S., Finn R.S., Abou-Alfa G.K., Cheng A.L., Yau T., Furuse J., Park J.W., Boyd Z. (2020). Association of inflammatory biomarkers with clinical outcomes in nivolumab-treated patients with advanced hepatocellular carcinoma. J. Hepatol..

[14] Le D.T., Uram J.N., Wang H., Bartlett B.R., Kemberling H., Eyring A.D., Skora A.D., Luber B.S., Azad N.S., Laheru D. (2015). PD-1 Blockade in Tumors with Mismatch-Repair Deficiency. N. Engl. J. Med..

[15] Harding J.J., Nandakumar S., Armenia J., Khalil D.N., Albano M., Ly M., Shia J., Hechtman J.F., Kundra R., El Dika I. (2019). Prospective Genotyping of Hepatocellular Carcinoma: Clinical Implications of Next-Generation Sequencing for Matching Patients to Targeted and Immune Therapies. Clin. Cancer Res. Off. J. Am. Assoc. Cancer Res..

[16] Mitsuhashi N., Kobayashi S., Doki T., Kimura F., Shimizu H., Yoshidome H., Ohtsuka M., Kato A., Yoshitomi H., Nozawa S. (2008). Clinical significance of alpha-fetoprotein: Involvement in proliferation, angiogenesis, and apoptosis of hepatocellular carcinoma. J. Gastroenterol. Hepatol..

[17] Shao Y.Y., Liu T.H., Hsu C., Lu L.C., Shen Y.C., Lin Z.Z., Cheng A.L., Hsu C.H. (2019). Early alpha-foetoprotein response associated with treatment efficacy of immune checkpoint inhibitors for advanced hepatocellular carcinoma. Liver Int. Off. J. Int. Assoc. Study Liver.

[18] Hsu W.F., Chuang P.H., Chen C.K., Wang H.W., Tsai M.H., Su W.P., Chen H.Y., Yang C.Y., Lin C.C., Huang G.T. (2020). Predictors of response and survival in patients with unresectable hepatocellular carcinoma treated with nivolumab: Real-world experience. Am. J. Cancer Res..

[19] Lee P.C., Chao Y., Chen M.H., Lan K.H., Lee C.J., Lee I.C., Chen S.C., Hou M.C., Huang Y.H. (2020). Predictors of Response and Survival in Immune Checkpoint Inhibitor-Treated Unresectable Hepatocellular Carcinoma. Cancers.

[20] Tian B.W., Yan L.J., Ding Z.N., Liu H., Meng G.X., Xue J.S., Han C.L., Dong Z.R., Hong J.G., Chen Z.Q. (2023). Early alpha-fetoprotein response predicts prognosis of immune checkpoint inhibitor and targeted therapy for hepatocellular carcinoma: A systematic review with meta-analysis. Expert Rev. Gastroenterol. Hepatol..

[21] (2018). EASL Clinical Practice Guidelines: Management of hepatocellular carcinoma. J. Hepatol..

[22] Lencioni R., Llovet J.M. (2010). Modified RECIST (mRECIST) assessment for hepatocellular carcinoma. Semin. Liver Dis..

[23] Kim H.I., Lim J., Shim J.H. (2021). Role of the alpha-fetoprotein response in immune checkpoint inhibitor-based treatment of patients with hepatocellular carcinoma. J. Cancer Res. Clin. Oncol..

[24] Pinato D.J., Mauri F.A., Spina P., Cain O., Siddique A., Goldin R., Victor S., Pizio C., Akarca A.U., Boldorini R.L. (2019). Clinical implications of heterogeneity in PD-L1 immunohistochemical detection in hepatocellular carcinoma: The Blueprint-HCC study. Br. J. Cancer.

[25] Topalian S.L., Taube J.M., Anders R.A., Pardoll D.M. (2016). Mechanism-driven biomarkers to guide immune checkpoint blockade in cancer therapy. Nat. Rev. Cancer.

[26] Hansen A.R., Siu L.L. (2016). PD-L1 Testing in Cancer: Challenges in Companion Diagnostic Development. JAMA Oncol..

[27] Galle P.R., Foerster F., Kudo M., Chan S.L., Llovet J.M., Qin S., Schelman W.R., Chintharlapalli S., Abada P.B., Sherman M. (2019). Biology and significance of alpha-fetoprotein in hepatocellular carcinoma. Liver Int. Off. J. Int. Assoc. Study Liver.

[28] Zhu A.X., Kang Y.K., Yen C.J., Finn R.S., Galle P.R., Llovet J.M., Assenat E., Brandi G., Pracht M., Lim H.Y. (2019). Ramucirumab after sorafenib in patients with advanced hepatocellular carcinoma and increased α-fetoprotein concentrations (REACH-2): A randomised, double-blind, placebo-controlled, phase 3 trial. Lancet Oncol..

[29] Peng S.Y., Chen W.J., Lai P.L., Jeng Y.M., Sheu J.C., Hsu H.C. (2004). High alpha-fetoprotein level correlates with high stage, early recurrence and poor prognosis of hepatocellular carcinoma: Significance of hepatitis virus infection, age, p53 and beta-catenin mutations. Int. J. Cancer.

[30] Terentiev A.A., Moldogazieva N.T. (2013). Alpha-fetoprotein: A renaissance. Tumour Biol. J. Int. Soc. Oncodev. Biol. Med..

[31] Shan Y.F., Huang Y.L., Xie Y.K., Tan Y.H., Chen B.C., Zhou M.T., Shi H.Q., Yu Z.P., Song Q.T., Zhang Q.Y. (2011). Angiogenesis and clinicopathologic characteristics in different hepatocellular carcinoma subtypes defined by EpCAM and α-fetoprotein expression status. Med. Oncol..

[32] Fukumura D., Kloepper J., Amoozgar Z., Duda D.G., Jain R.K. (2018). Enhancing cancer immunotherapy using antiangiogenics: Opportunities and challenges. Nat. Rev. Clin. Oncol..

[33] Li X., Sun W., Ding X., Li W., Chen J. (2022). Prognostic model of immune checkpoint inhibitors combined with anti-angiogenic agents in unresectable hepatocellular carcinoma. Front. Immunol..

[34] Lu L.C., Hsu C., Shao Y.Y., Chao Y., Yen C.J., Shih I.L., Hung Y.P., Chang C.J., Shen Y.C., Guo J.C. (2019). Differential Organ-Specific Tumor Response to Immune Checkpoint Inhibitors in Hepatocellular Carcinoma. Liver Cancer.

[35] Scheiner B., Pomej K., Kirstein M.M., Hucke F., Finkelmeier F., Waidmann O., Himmelsbach V., Schulze K., von Felden J., Fründt T.W. (2022). Prognosis of patients with hepatocellular carcinoma treated with immunotherapy—Development and validation of the CRAFITY score. J. Hepatol..

[36] Dharmapuri S., Özbek U., Lin J.Y., Sung M., Schwartz M., Branch A.D., Ang C. (2020). Predictive value of neutrophil to lymphocyte ratio and platelet to lymphocyte ratio in advanced hepatocellular carcinoma patients treated with anti-PD-1 therapy. Cancer Med..

[37] Zhang Y., Lu L., He Z., Xu Z., Xiang Z., Nie R.C., Lin W., Chen W., Zhou J., Yin Y. (2022). C-Reactive Protein Levels Predict Responses to PD-1 Inhibitors in Hepatocellular Carcinoma Patients. Front. Immunol..

[38] Guthrie G.J., Charles K.A., Roxburgh C.S., Horgan P.G., McMillan D.C., Clarke S.J. (2013). The systemic inflammation-based neutrophil-lymphocyte ratio: Experience in patients with cancer. Crit. Rev. Oncol. Hematol..

[39] Michael M., Goldstein D., Clarke S.J., Milner A.D., Beale P., Friedlander M., Mitchell P. (2006). Prognostic factors predictive of response and survival to a modified FOLFOX regimen: Importance of an increased neutrophil count. Clin. Color. Cancer.

[40] Mantovani A., Allavena P., Sica A., Balkwill F. (2008). Cancer-related inflammation. Nature.

[41] Diakos C.I., Charles K.A., McMillan D.C., Clarke S.J. (2014). Cancer-related inflammation and treatment effectiveness. Lancet Oncol..

[42] Wu Y.L., Fulgenzi C.A.M., D’Alessio A., Cheon J., Nishida N., Saeed A., Wietharn B., Cammarota A., Pressiani T., Personeni N. (2022). Neutrophil-to-Lymphocyte and Platelet-to-Lymphocyte Ratios as Prognostic Biomarkers in Unresectable Hepatocellular Carcinoma Treated with Atezolizumab plus Bevacizumab. Cancers.

[43] Hsu W.F., Lai H.C., Chen C.K., Wang H.W., Chuang P.H., Tsai M.H., Chen S.H., Chu C.S., Su W.P., Chou J.W. (2023). Combined CRAFITY score and α-fetoprotein response predicts treatment outcomes in patients with unresectable hepatocellular carcinoma receiving anti-programmed death-1 blockade-based immunotherapy. Am. J. Cancer Res..

[44] Chen Q., Deng Y., Zhao C., Huang Z., Zhang W., Yang Y., Bai Y., Tu J., Li B., Wu W. (2023). Nomogram for tumour response based on prospective cohorts of hepatocellular carcinoma patients receiving immunotherapy combined with targeted therapy: Development and validation. Ann. Transl. Med..

[45] Ma D., Liu M., Zhai X., Li X., Jin B., Liu Y. (2023). Development and validation of prognostic risk prediction models for hepatocellular carcinoma patients treated with immune checkpoint inhibitors based on a systematic review and meta-analysis of 47 cohorts. Front. Immunol..

[46] Zhang L., Feng J., Kuang T., Chai D., Qiu Z., Deng W., Dong K., Zhao K., Wang W. (2023). Blood biomarkers predict outcomes in patients with hepatocellular carcinoma treated with immune checkpoint Inhibitors: A pooled analysis of 44 retrospective sudies. Int. Immunopharmacol..

