# Supplementary material for: Development and Assessment of Nomogram Based on AFP Response for Patients with Unresectable Hepatocellular Carcinoma Treated with Immune Checkpoint Inhibitors
Source: Cancers (Basel). 2023 Oct 25;15(21):5131. doi: 10.3390/cancers15215131 (PMC10647527; doi:10.3390/cancers15215131)
Supplement: Supplementary file 1 [file cancers-15-05131-s001.zip › cancers-2577295-supplementary.pdf]

Supplementary Materials

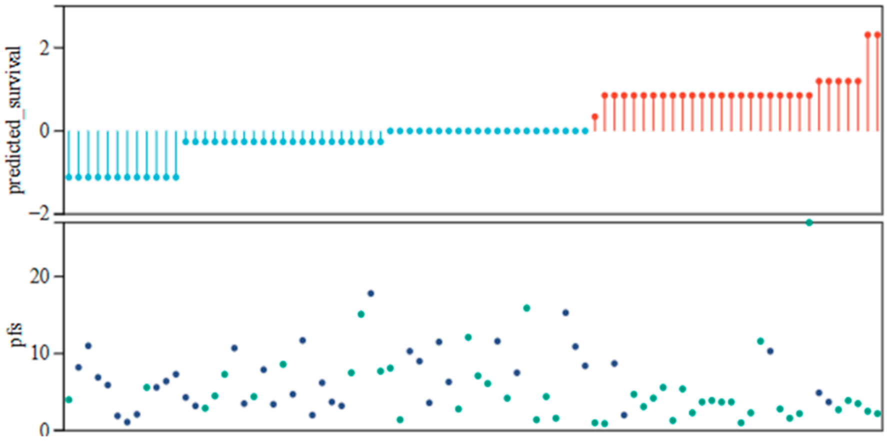

Figure S1. The mPFS distribution in the training set.

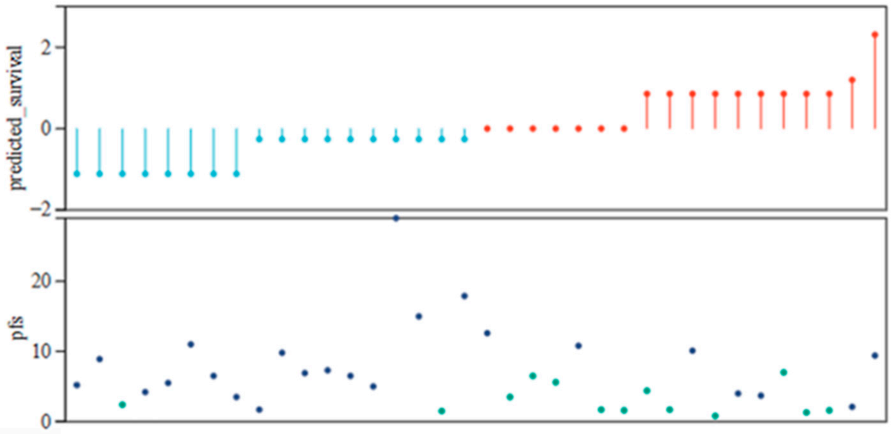

Figure S2. The mPFS distribution in the validation set.
